# Radiomics performance to identify high-risk carotid artery plaques: a systematic review

**DOI:** 10.1186/s41747-026-00843-7

**Published:** 2026-11-03

**Authors:** Jessica D’lima, François Rheault, Ann-Marie Beaudoin

**Affiliations:** 1 Department of Medicine, University of Melbourne, Parkville, VIC Australia; 2 Sherbrooke Connectivity and Imaging Lab (SCIL), Faculty of Sciences, University of Sherbrooke, Sherbrooke, QC Canada; 3 Department of Medicine, Faculty of Medicine and Health Sciences, University of Sherbrooke, Sherbrooke, QC Canada; 4 Centre de Recherche du Centre Hospitalier Universitaire de Sherbrooke, University of Sherbrooke, Sherbrooke, QC Canada

**Keywords:** Atherosclerotic plaque, Carotid stenosis, Ischemic stroke, Radiomics, Risk assessment

## Abstract

**Background:**

Stroke is a leading cause of mortality worldwide, with 15–20% of ischemic strokes caused by extracranial carotid atherosclerotic disease. Risk stratification has traditionally relied on luminal stenosis severity, which inadequately reflects plaque vulnerability. Radiomics offers an emerging solution by extracting high-dimensional quantitative data from standard medical images. This systematic review evaluated the performance of radiomics-based models for identifying high-risk carotid plaques.

**Materials and methods:**

A systematic search was conducted across multiple databases. Studies were included if they developed or validated radiomics-based models for carotid plaque assessment and reported performance metrics, specifically the area under the curve (AUC). Data were extracted on study design, imaging modalities, segmentation methods, and modeling techniques.

**Results:**

Twenty-eight studies involving 6,108 patients (72.4% male) were included. Computed tomography angiography was the most commonly used imaging modality (*n* = 16), and manual region-of-interest segmentation was performed in 27 of 28 studies. Radiomics models showed strong predictive performance. Internal validation AUCs were as high as 0.984, whereas combined models incorporating radiomics with clinical and/or conventional radiological features achieved the best predictive accuracy, often exceeding 0.90. However, only four studies included external validation cohorts.

**Conclusion:**

Radiomics is a powerful, non-invasive tool for identifying high-risk carotid plaques, offering superior discriminative power over conventional stenosis-based assessments. Despite these promising results, the field faces significant hurdles, and future research must prioritize prospective multicenter trials, the development of automated segmentation workflows, and the correlation of radiomic features with histopathological findings to ensure clinical reproducibility and transition into routine stroke prevention.

**Key Points:**

***Question*** Standard carotid atherosclerotic disease assessments fail to capture biological plaque complexity and vulnerability, necessitating objective, reproducible, and non-invasive methods to improve stroke prevention.

***Findings*** Radiomics and combined models demonstrate superior capability in identifying high-risk carotid plaques compared to traditional assessments. With four external validations, the field remains exploratory.

***Relevance statement*** Radiomics offers a non-invasive tool for personalized stroke prevention. However, technical barriers currently prevent its transition to routine clinical use, including reliance on manual segmentation and low compliance with international standardization guidelines.

**Graphical Abstract:**

**Figure:**
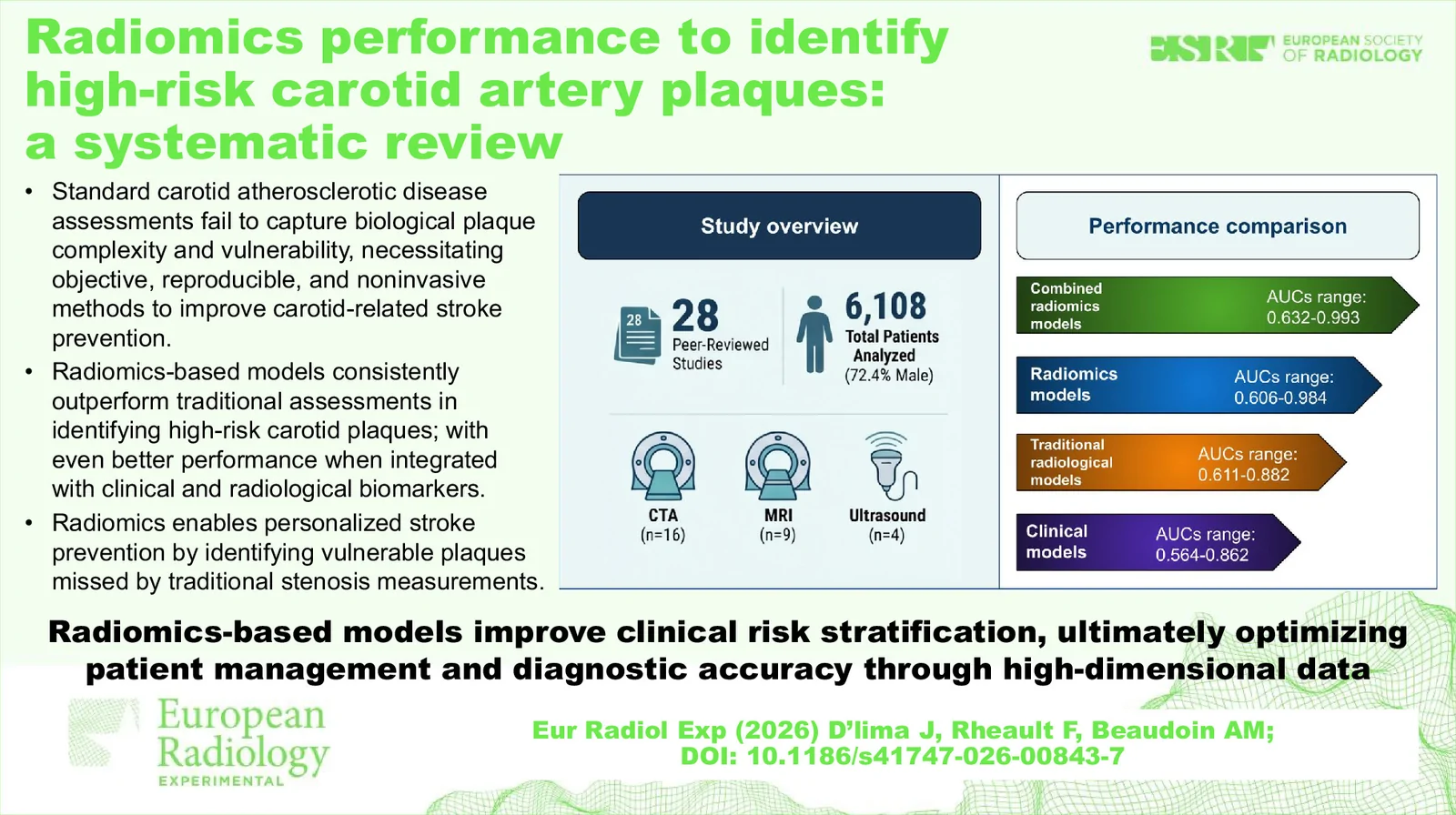

## Introduction

Stroke remains one of the leading causes of mortality and long-term disability worldwide. The most recent and comprehensive epidemiological analyses, including the Global Burden of Disease Study 2021, report that ischemic stroke constituted approximately 65% of all incident strokes worldwide in 2021, with some regional variation [1]. Despite major advances in acute reperfusion therapies and secondary prevention strategies, the incidence of ischemic stroke remains high, particularly in aging populations and among individuals with cardiovascular risk factors. Accurate identification of the underlying etiological mechanisms of ischemic stroke, therefore, represents a critical step toward improving risk stratification and optimizing preventive strategies.

Atherosclerotic disease of the extracranial carotid arteries is a major cause of ischemic stroke, accounting for an estimated 15–20% of cases, depending on study populations and etiological classifications [2]. Traditionally, the embolic risk associated with carotid artery disease has been primarily assessed based on the degree of luminal stenosis, with stenosis severity defined according to the angiographic method used in the North American Symptomatic Carotid Endarterectomy Trial (NASCET) [3]. However, growing evidence suggests that stroke risk is not solely determined by the severity of luminal narrowing but also by the biological and morphological characteristics of the atherosclerotic plaque. Features such as lipid-rich necrotic core, inflammation, neovascularization, intraplaque hemorrhage, and fibrous cap thinning have been associated with increased plaque vulnerability and embolic potential, sometimes independently of the degree of stenosis [4, 5].

Conventional radiological assessment of carotid atherosclerosis has important limitations. In routine clinical practice, evaluation largely relies on the percentage of luminal stenosis, which fails to capture the biological complexity of atherosclerotic plaques [6]. Qualitative assessment of plaque vulnerability features, when performed, requires specialized expertise, is time-consuming, and remains subject to significant inter- and intra-observer variability. Furthermore, access to advanced vascular imaging expertise is heterogeneous across centers, limiting reproducibility and generalizability. These constraints underscore the need for objective, automated, and reproducible methods to provide a more comprehensive assessment of carotid plaque-related stroke risk.

Radiomics is an emerging approach that aims to extract a large number of quantitative features from standard medical images, capturing information related to tissue intensity, texture, shape, and spatial heterogeneity [7]. Many of these features are invisible to the human eye, and radiomics enables the transformation of medical images into high-dimensional, mineable data. When combined with machine learning techniques, radiomics has demonstrated promising results across multiple fields, including oncology, cardiology, and neurology, for disease classification, prognostication, and treatment response prediction [8–10].

**Fig. 1:**
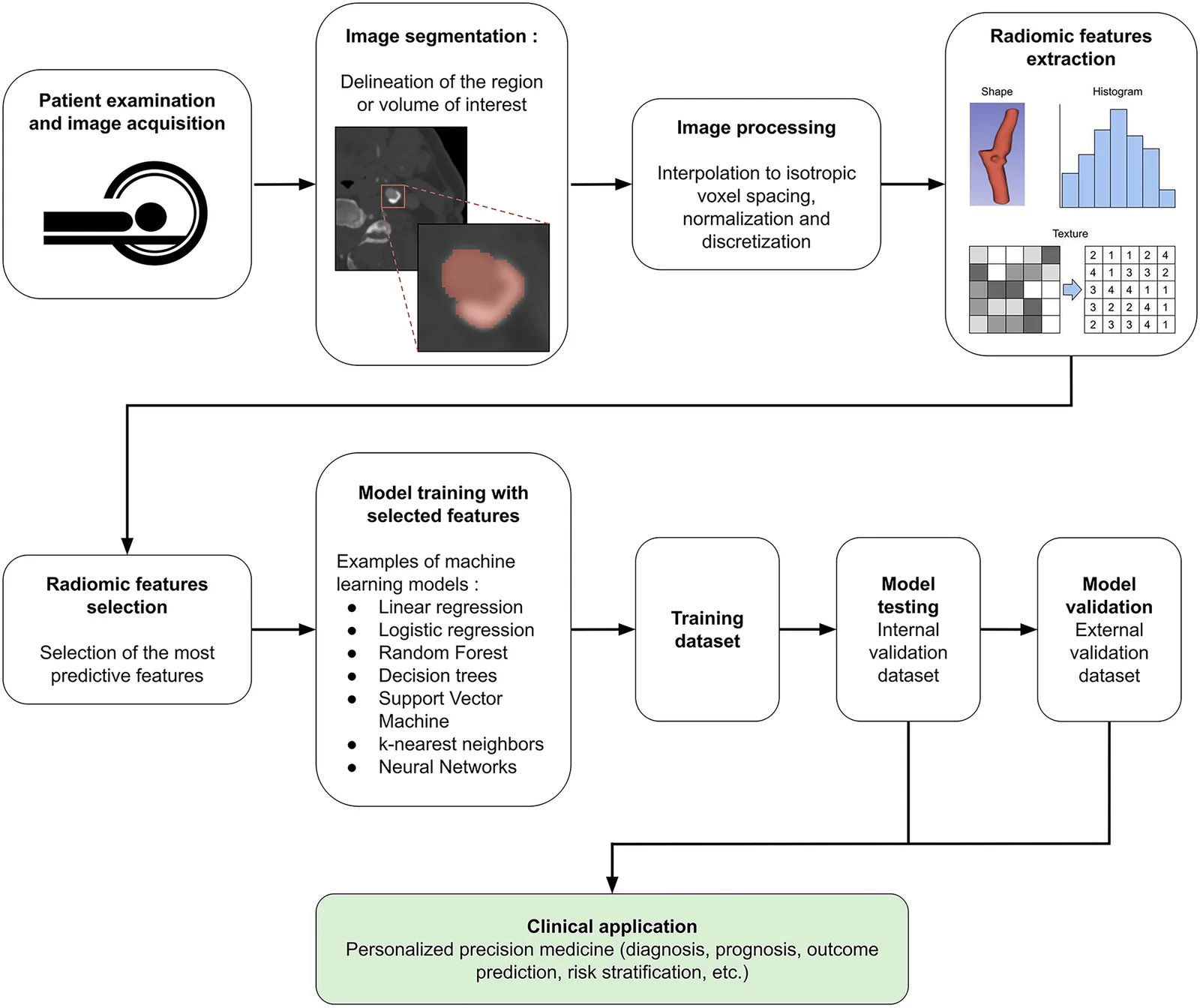
Radiomics workflow. After clinical assessment of the patient and image acquisition and reconstruction, the region or volume of interest is identified and segmented. In this example, the symptomatic carotid artery at the level of the carotid bifurcation (intraluminal space and plaque) was manually segmented. Image processing then includes various steps, including interpolation, normalization and discretization. Radiomics features are then extracted from the region or volume of interest, and the most predictive features are selected to train machine learning models to make clinical predictions, such as stroke recurrence. The best-performing models are then tested on a different dataset (an internal validation set that was not seen during training). Finally, the external validation is achieved with a new dataset coming from an external source (*e.g*., another scanner from another hospital)

Applied to carotid artery imaging, radiomics offers a unique opportunity to non-invasively characterize plaque composition and heterogeneity. By integrating machine learning algorithms, radiomics-based approaches allow the development of predictive models capable of identifying imaging signatures associated with plaque vulnerability and stroke risk (Fig. 1). Several recent studies have investigated radiomics using different imaging modalities, including ultrasound (US), computed tomography angiography (CTA), and magnetic resonance imaging (MRI), reporting encouraging improvements in risk stratification compared with conventional imaging metrics. However, substantial methodological heterogeneity across studies, including differences in imaging protocols, segmentation strategies, feature extraction pipelines, and machine learning algorithms, limits direct comparison and interpretation of reported performance.

The objective of this systematic review is to summarize the existing literature on radiomics-based assessment of carotid plaques, documenting the diversity of modeling approaches (including single-modality and combined models) and their reported performance across various imaging modalities.

## Materials and methods

### Literature search strategy

This review was performed according to Preferred Reporting Items for Systematic Review and Meta-Analyses (PRISMA) guidelines [11]. The completed PRISMA checklist is included in the Supplemental Materials. The protocol of this systematic review was registered with the International Prospective Register of Systematic Reviews (PROSPERO, Registration No. CRD42024565750). The literature search strategy was developed in collaboration with two experienced health sciences librarians from the University of Sherbrooke and was designed to comprehensively identify studies evaluating radiomics- and machine-learning-based approaches for assessing carotid atherosclerotic disease.

While the initial protocol only specified PubMed and Medline, the search was expanded to five databases (PubMed, Embase, Scopus, the Cochrane Library, and Google Scholar) to ensure a more comprehensive capture of the rapidly evolving literature on radiomics and to minimize selection bias. A systematic search was conducted using controlled vocabulary (*e.g*., MeSH and Emtree terms) and free-text keywords related to radiomics, texture analysis, artificial intelligence, machine learning, and deep learning, as well as terms describing carotid stenosis, carotid artery disease, atherosclerotic plaque, and plaque vulnerability. Database-specific search syntaxes were applied to optimize sensitivity. In addition, reference lists of relevant reviews were manually screened to identify additional eligible studies. The search details in each database are outlined in Supplemental Materials.

### Study selection and data extraction

Both prospective and retrospective studies were considered eligible if they met the following inclusion criteria: (i) participants were patients with carotid artery plaque and/or stenosis; (ii) the study reported the development and/or validation of a diagnostic or prognostic model based on radiomics; (iii) outcomes included model performance metrics; and (iv) the study was a peer-reviewed scientific report published in English or French up to the date of the literature search. Eligibility assessment was performed by investigators fluent in both languages, and all selected studies were published in English-language peer-reviewed journals.

Studies were excluded if they involved non-human participants; focused exclusively on deep learning approaches without the inclusion of radiomics features (RFs); or were published as letters, reviews, case reports, abstracts, editorials, or other non-original research formats. Studies that assessed perivascular adipose tissue (PVAT) as the sole region of interest (ROI) were excluded, whereas studies that analyzed RFs from both the carotid plaque and the surrounding PVAT were considered eligible. To ensure a degree of homogeneity across the studied populations, we excluded studies that focused exclusively on specific patient subgroups. Therefore, studies limited to patients with type 2 diabetes, carotid stents, or intracranial stenosis were excluded. Finally, some studies relied solely on univariate analysis (such as the Mann-Whitney U test or independent t-test) to assess a single factor’s relationship with stroke risk. In radiomics, the goal is often to extract hundreds of features that, when combined into a multivariate model, can capture the biological complexity of a plaque better than any single marker alone. Because this review focuses on the performance of these integrated models, reports that did not move beyond testing individual features were excluded.

Two reviewers (A-M.B. and J.D.) independently screened titles and abstracts for initial eligibility, followed by full-text review to determine final inclusion. The extraction process was also performed and verified by both authors to mitigate potential bias. Specifically, data were extracted by one reviewer into a standardized template and independently verified by the second reviewer to ensure accuracy and completeness. Any discrepancies were resolved through discussion and consensus. For each included study, data were systematically extracted on study characteristics (publication year, country, study design, purpose), population details (total number of patients, number of symptomatic *versus* asymptomatic subjects, sex distribution, mean age, and cardiovascular comorbidities including hypertension, diabetes mellitus, dyslipidemia, and smoking), and carotid disease characteristics (degree of stenosis when reported and method of stenosis assessment). Imaging-related data included imaging modalities, type of features used (RFs, conventional radiological assessment, clinical variables, or combinations thereof), ROI, segmentation methods, image filters, and RF extraction software. Model-related information included the modeling approach, the number of features in the final model, the use of external validation, the primary study objective, and predictive performance.

The area under the receiver operating characteristic curve (AUC) was selected as the primary performance metric because it provides a reliable summary of a model’s discriminative performance (AUC of 1.00 denotes perfect separation) that remains robust across all decision thresholds. By using this standardized measure, it is possible to objectively synthesize the performance of radiomics models relative to traditional clinical and radiological assessments across the diverse study designs included in this review. To benchmark the technology's maximum current capability, the model with the highest AUC in the testing/validation cohort was selected for primary analysis. However, to mitigate performance-selection bias, we also extracted the AUC of all the utilized radiomics models to ensure a transparent representation of the evidence (Table S4).

### Statistical analysis

The statistical synthesis of this systematic review summarizes study and population characteristics using descriptive statistics, with continuous variables reported as means ± standard deviations or medians with interquartile ranges, and categorical data as frequencies and percentages. The analysis then focuses on the diagnostic and predictive performance of the models, utilizing the AUC to compare radiomics-based approaches with clinical and conventional radiological models.

The validation level of the included studies was categorized according to the Transparent Reporting of a Multivariable Prediction Model for Individual Prognosis or Diagnosis (TRIPOD) framework [12], a reporting guideline that assesses the completeness and transparency of studies describing the development and validation of prediction models. The Radiomics Quality Score (RQS) was utilized to evaluate the quality and reproducibility of radiomics studies [13]. This standardized assessment tool evaluates the methodological quality, transparency, and potential clinical applicability of radiomics studies across the entire workflow, from image acquisition and feature extraction to model validation and reproducibility.

As mentioned in the registered PROSPERO protocol, the original intent was to perform a meta-analysis. However, upon full-text review and data extraction, substantial methodological and technical heterogeneity among the included studies was identified. It was concluded that pooling these results into a single quantitative meta-analysis could lead to misleading conclusions about the technology’s predictive value.

## Results

### Literature assessment

**Fig. 2:**
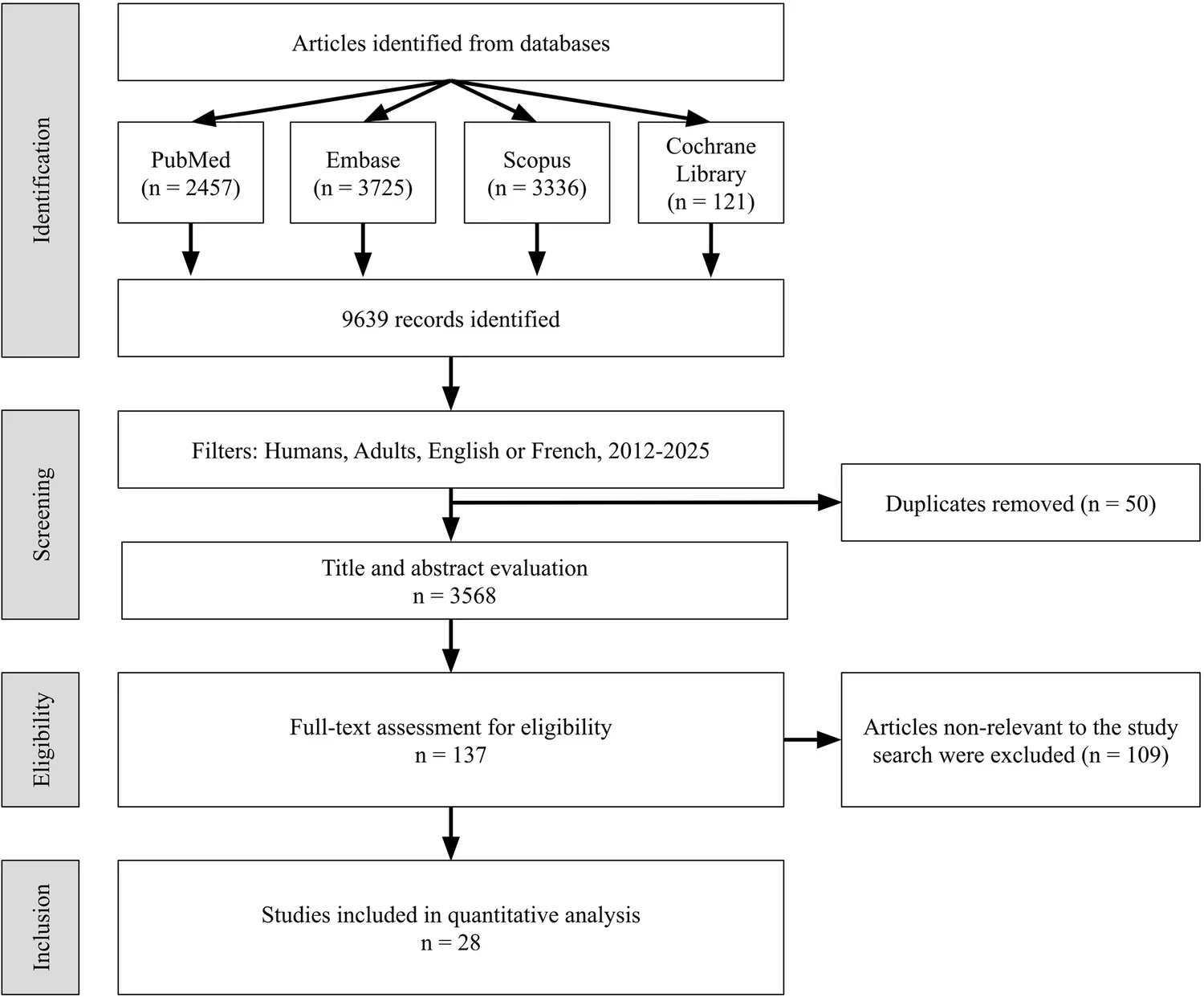
Flowchart of study selection. Articles were identified across multiple databases, and filters were applied when available. Studies published in English or French were included, as the investigators are fluent in both languages. A time filter (2012–2025) was applied because the concept of radiomics was first introduced in the literature in 2012

The database search identified a total of 9,639 records. After applying predefined filters (humans, adults, English or French language, publications from January 1st, 2012, to November 1st, 2025) and removing duplicates, 3,568 records remained for screening. Title and abstract screening resulted in 137 potentially eligible articles. Following full-text assessment, 109 articles were excluded due to a lack of relevance to the systematic review’s objectives. A total of 28 studies met the inclusion criteria and were included in the final quantitative analysis [14–41]. Figure 2 presents a flowchart of the study selection process.

### Study characteristics and patient population

The vast majority of studies included in this systematic review originate from China (*n* = 24), followed by the United Kingdom (*n* = 2), Italy (*n* = 1), and the United States (*n* = 1). Regarding study design, 23 were retrospective cohort studies, 2 were prospective, and 3 used a bidirectional cohort design. The combined study population comprised 6,108 patients, including 2,231 symptomatic and 3,726 asymptomatic cases. The discrepancy of 151 is attributable to the reporting methods of four specific studies. Three studies did not classify their participants into symptomatic or asymptomatic groups [15, 17, 39], for a total of 179 patients. In the study by Dong et al, the authors report data on 148 carotid arteries (34 symptomatic and 114 asymptomatic), whereas the actual cohort consisted of only 120 patients. This results in a differential of 28 when comparing carotid-based symptom counts to the unique patient count in that study.

The demographic distribution showed a predominance of males (72.4%) over females (28.3%), with mean or median ages across the cohorts generally ranging from the early 50s to mid-70s. Clinical comorbidities were frequent among the participants, with hypertension being the most prevalent (*n* = 3,234), followed by dyslipidemia (*n* = 2,417), diabetes mellitus (*n* = 2,281), and smoking (*n* = 1,882). The general characteristics of the included studies are summarized in the Table 1, and the specific aim of each study is indicated in Table S1.

**Table 1:** General characteristics of the included studies

Study	Country	Design (R, P, B)	Total *N*	Sx cases	Asx cases	Age (mean or median)	Males (*N*)	Females (*N*)	Hypertension (*N*)	Diabetes (*N*)	Dyslipidemia (*N*)	Smoking (*N*)	% Stenosis included	Detection of stenosis
Chen, Chao et al [14]	China	R	144	60	84	70.93 ± 9.10	110	34	111	52	69	65	30–99	CTA
Chen, Sihan et al [15]	China	R	115	75	40	51.2 ± 13.8	91	24	89	41			30–69	US
Cilla, Savino et al [16]	Italy	R	30			72.96 (50–86)	19	11	28	9	17		70–99	No mention
Dong, Zheng et al [17]	China	R	120	34	114	66.68 ± 7.75	100	20	97	47	47	39	50–99	CTA
Ebrahimian, Shadi et al [18]	USA	R	85			73 ± 10	56	29					< 50, 50–70, > 70	CTA
Guan, Le et al [19]	China	B	110	110	0	65.3 ± 13/63.3 ± 11.6	73	37	61	39		42		US
Gui, Chengzhi et al [20]	China	R	104	74	30	64.61 ± 7.46/64.90 ± 7.31	86	18					30–69	No mention
Han, Ling et al [21]	China	R	188	78	110	65.4	157	31					70–99	CTA
Han, Na et al [22]	China	R	127	60	67	60.55 ± 9.72	103	24	82	43	36			MRI
Hu, Weiming et al [23]	China	R	416	131	285	73.13 ± 9.12 (multiple groups)	304	112	284	115	35	86	30–99	CTA
Huang, Zhe et al [24]	China	P	548	381	167	63 ± 10	373	175	273	245	360	208		US
Huang, Zhe et al [25]	China	B	2009	0	2009	64	1333	676	855	967	1115	645		US
Lao, Qun et al [26]	China	R	107	107	0	65.8 ± 3.8/66.1 ± 3.7	97	10	85	34	41	33	70–99	CTA
Le, Elizabeth P. V. et al [27]	UK	R	41	41	0	74.1 ± 8.4	32	9	27	8		29		
Le, Elizabeth P. V. et al [28]	UK	R	66	41	25	71.4 ± 9.2	50	16	39	11	49	45		
Li, Zhi-Li et al [29]	China	R	123	64	59	71.86 ± 7.13/70.06 ± 9.46	85	38	99	53	43	46	≥ 1%	CTA
Liu, M et al [30]	China	R	280	141	139	63.8 ± 7.2 (multiple groups)	201	79	209	99	327	202		CTA
Meddings, Zakaria et al [31]	China (patients)	R	55	55	0	62.6 ± 10.6	35	20	36		36	26	≥ 50%	MRI
Shan, Dezhi et al [32]	China	R	74	44	30	66.9 ± 8.82	63	11	52	29		41		CTA or US
Shi, Jinglong et al [33]	China	R	167	70	97	66.2 ± 7.7	131	36	115	48	73	91		CTA
Song, Jiajia et al [34]	China	R	202	202	0	50.3 ± 2.1/62.9 ± 1.7	99	103	16	112	19			US
Wang, Lingjie et al [35]	China	R	193	123	70	64.06 ± 8.76/63.7 ± 9.87	157	36	147	76		87		CTA
Xia, Hai et al [36]	China	R	179	34	145	65.4	153	66	117	45		46	30–50	CTA
Yu, Fan et al [37]	China	P	159	0	159	64.97 ± 7.76	137	22	126	71	37	31		US
Zhang, Lianlian et al [38]	China	B	150	150	0	61.7 ± 10.0	120	30	109	53	39	39		US
Zhang, Ranying et al [39]	China	R	162	108	54	66.8 ± 7.37	148	14	124	56	74	53	30–99	CTA or US
Zhang, Shuai et al [40]	China	R	64			60.7 ± 10.4	49	15						CTA
Zhang, Xun et al [41]	China	R	90	48	42	58.43 ± 10.62/59.27 ± 11.16	58	32	53	28		28	50	MRI

*Asx* Asymptomatic, *B* Bidirectional, *CTA* Computed tomography angiography, *MRI* Magnetic resonance imaging, *N* Number, *P* Prospective, *R* Retrospective, *Sx* Symptomatic, *US* Ultrasonography

### Imaging modalities and stenosis assessment

In terms of diagnostic modalities, CTA was the most frequently utilized imaging tool (*n* = 16, including three dual-energy studies), followed by MRI (*n* = 9, including five high-resolution MRI studies) and US (*n* = 4). Details regarding imaging protocols and acquisition parameters for CTA- and MRI-based studies are provided in Tables S5 and S6. The ROI typically targeted the carotid plaques, though specific definitions varied from the entire 3D plaque volume to the single slice with the largest plaque area. Some studies expanded their ROI to include the carotid artery wall or perivascular adipose tissue (Table S2). For the assessment of carotid disease severity, the NASCET method [3] was the most frequent standard for measuring stenosis (*n* = 15), although the degree of stenosis was not explicitly mentioned in 14 of the studies. When reported, inclusion criteria for stenosis typically ranged from 30% to 99%.

### Image segmentation and feature extraction

Segmentation was almost always performed manually (*n* = 27), with only one study utilizing an automated approach (Table S2). Multi-slice (3D) segmentation was more common (*n* = 16) than single-slice (2D) analysis (*n* = 9), and three studies utilized both methods. Researchers frequently applied image filters, most notably Wavelet (*n* = 18) and Gaussian (*n* = 13) filters. All 28 studies extracted radiomic features, including shape, first-order statistics, and various texture matrices such as the gray-level co-occurrence matrix (GLCM), gray-level dependence matrix (GLDM), gray-level size zone matrix (GLSZM), gray-level run length matrix (GLRLM), and neighborhood gray-tone difference matrix (NGTDM). Many studies integrated these with other data types. Regarding software, PyRadiomics was the most widely used platform for feature extraction (*n* = 17). Only five studies explicitly confirmed compliance with the Image Biomarker Standardization Initiative (IBSI), an international effort to standardize radiomic feature extraction by providing consensus definitions, benchmark datasets, reference values, and reporting guidelines to improve reproducibility across studies [42].

The traditional radiological models utilized established visual and quantitative metrics to identify high-risk plaque features, primarily focusing on luminal geometry, plaque morphology, and compositional signs of vulnerability. The most universal component was the degree of luminal stenosis, typically measured using the NASCET method [3]. Models frequently included physical dimensions of the plaque to gauge its severity, including plaque thickness, plaque length and volume, plaque burden (proportion of the vessel occupied by the plaque), and growth mode (concentric *versus* eccentric). Some traditional models were also defined by the presence of specific high-risk components identified through visual inspection, such as intraplaque hemorrhage, lipid-rich necrotic core, calcifications, plaque ulceration (defined as contrast extension into the plaque), fibrous cap status (thin, ruptured, or intact), and plaque enhancement (degree of contrast uptake within the plaque).

The clinical models were designed to capture a comprehensive profile of patient health, focusing on demographics (*e.g*., age, sex, body mass index), modifiable risk factors (*e.g*., smoking, alcohol consumption), medical comorbidities (*e.g*., hypertension, diabetes mellitus, dyslipidemia, coronary artery disease, medication history), and specific laboratory biomarkers (*e.g*., lipid profiles, C-reactive protein). Some studies chose to utilize established clinical risk scores. Huang et al [25] used the Revised Framingham Stroke Risk Profile to classify baseline cardiovascular risk, while Guan et al [19] integrated the ABCD2 Score, which assesses age, blood pressure, clinical features, duration of symptoms, and diabetes, to predict the risk of recurrent transient ischemic attack or stroke.

### Modeling methods and validation

The Least Absolute Shrinkage and Selection Operator was the most common method for feature reduction and selection, utilized in 21 of the 28 included studies. Logistic regression was the most frequent modeling technique (15/21 studies reporting validation dataset results), followed by support vector machine (8/21), random forest (4/21) and extreme gradient boosting (3/21) (Table S4). The number of features in the final models was highly heterogeneous (ranging from 2 to 107). In the TRIPOD classification assessment (Table S7), 16 studies reached a Type 2 validation (internal validation via random split or cross-validation), and only four performed a Type 3 validation using independent institutional data. When assessed using the RQS (Table S8), the included studies obtained scores ranging from 9 to 16/36. Several items were not fulfilled in any of the included studies (items 3, 4, 11, 15, and 16). In contrast, some items were consistently well addressed (items 1, 5, 6, 9, and 14). The lack of external validation negatively impacted the overall RQS of most studies.

### Predictive performance

**Fig. 3:**
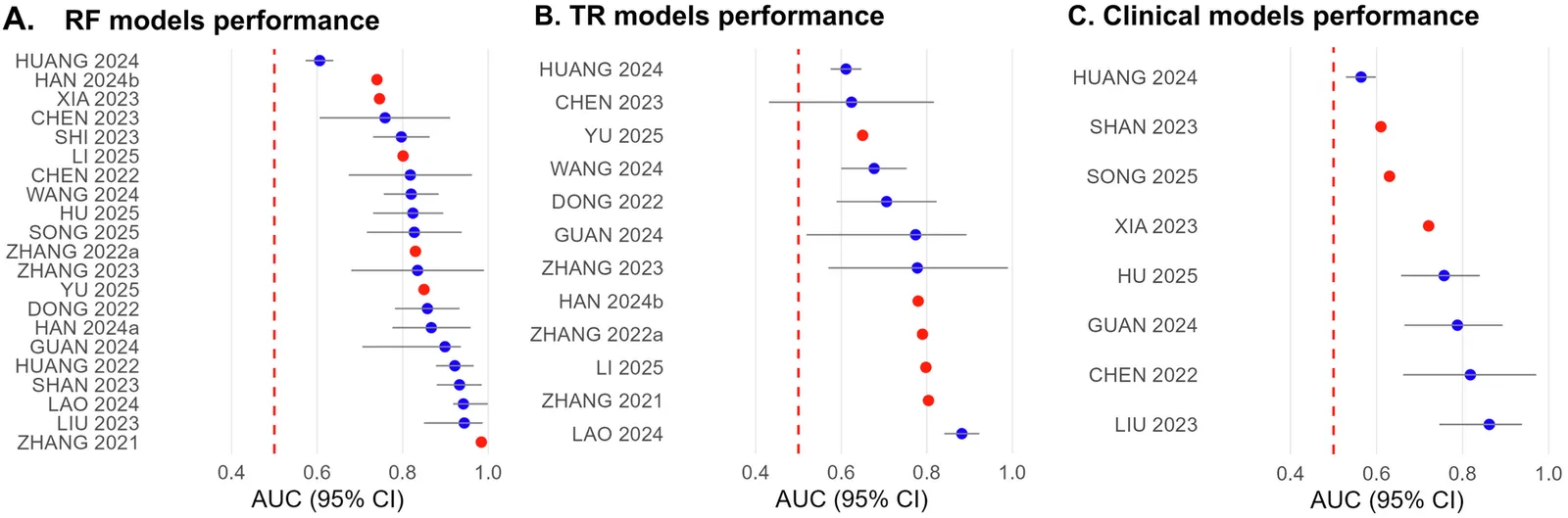
Performance (AUC) of the different models with the internal validation datasets. **A** presents the performance of radiomics feature (RF) models, **B** presents the performance of traditional radiological (TR) models, and **C** presents the performance of clinical models. AUC, area under the curve; CI, confidence interval; RF, radiomics features; TR, traditional radiological. Blue markers indicate AUC values with reported 95% CI, shown as horizontal lines; red markers indicate AUC values for which 95% CIs were not available

In the testing datasets (internal validation), radiomics models showed strong discriminative performance (Table 2). The reported AUC values for radiomics models spanned from 0.606 to 0.984 (Fig. 3), with several studies demonstrating high predictive accuracy exceeding 0.80. When compared, these models consistently showed higher AUCs than traditional radiological models, which ranged from 0.611 to 0.882. Clinical models demonstrated variable results, with performance peaks around 0.862, yet they frequently trailed the combined or radiomics-only approaches.

**Table 2:** Performance of the best predictive models in the included studies

Study	Testing set (internal validation)			External validation set		
	RF model AUC (95% CI)	TR model AUC (95% CI)	C model AUC (95% CI)	RF model AUC (95% CI)	TR model AUC (95% CI)	C model AUC (95% CI)
Chen, Chao et al [14]	0.759 (0.606–0.911)	0.624 (0.431–0.817)				
Chen, Sihan et al [15]	0.818 (0.674–0.962)		0.818 (0.662–0.971)			
Cilla, Savino et al [16]						
Dong, Zheng et al [17]	0.858 (0.782–0.933)	0.706 (0.589–0.823)				
Ebrahimian, Shadi et al [18]						
Guan, Le et al [19]	0.899 (0.706–0.936)	0.774 (0.519–0.893)	0.788 (0.665–0.893)			
Gui, Chengzhi et al [20]						
Han, Ling et al [21]	0.867 (0.776–0.959)					
Han, Na et al [22]	0.74	0.78				
Hu, Weiming et al [23]	0.824 (0.731–0.895)		0.757 (0.657–0.840)	0.785 (0.696–0.858)		0.743 (0.651–0.822)
Huang, Zhe et al [24]	0.922 (0.878–0.966)					
Huang, Zhe et al [25]	0.606 (0.573–0.638)	0.611 (0.575–0.647)	0.564 (0.529–0.598)	0.607 (0.534–0.680) 0.558 (0.414–0.701)	0.517 (0.436–0.598) 0.565 (0.455–0.675)	0.561 (0.483–0.640) 0.558 (0.491–0.626)
Lao, Qun et al [26]	0.942 (0.918–0.999)	0.882 (0.841–0.923)				
Le, Elizabeth P. V. et al [27]						
Le, Elizabeth P. V. et al [28]						
Li, Zhi-Li et al [29]	0.801	0.798				
Liu, M et al [30]	0.944 (0.850–0.987)		0.862 (0.746–0.938)	0.932 (0.857–0.975)		0.818 (0.721–0.892)
Meddings, Zakaria et al [31]						
Shan, Dezhi et al [32]	0.933 (0.880–0.985)		0.61			
Shi, Jinglong et al [33]	0.797 (0.731–0.863)					
Song, Jiajia et al [34]	0.827 (0.716–0.938)		0.63			
Wang, Lingjie et al [35]	0.82 (0.756–0.884)	0.677 (0.600–0.753)				
Xia, Hai et al [36]	0.746		0.721			
Yu, Fan et al [37]	0.85	0.65				
Zhang, Lianlian et al [28]	0.83	0.79				
Zhang, Ranying et al [39]	0.984	0.804				
Zhang, Shuai et al [40]				0.725 (0.562–0.888)		0.761 (0.596–0.927)
Zhang, Xun et al [41]	0.835 (0.68–0.99)	0.778 (0.57–0.99)				

*AUC* Area under the curve, *C* Clinical, *CI* Confidence interval, *RF* Radiomics features, *TR* Traditional radiological

In the external validation cohorts, radiomics models continued to demonstrate moderate to high discriminative performance (Table 2). Notably, the highest recorded external performance reached an AUC of 0.932 for a radiomics model, outperforming the corresponding clinical model at 0.818 [30]. However, performance was not uniformly superior, as evidenced by a study in which the clinical model (AUC 0.761) slightly outperformed the radiomics model (AUC 0.725) [40].

Some studies created combined models to enhance predictive performance in discriminating high-risk carotid plaques (Table 3). A total of eight studies added traditional radiological metrics to RFs, with AUCs ranging from 0.82 to 0.99. A combined radiomics and clinical model was used in five studies, also showing good performance (AUCs between 0.804 and 0.977). Models that combined conventional measures (radiological and clinical data) were used in only two studies, with moderate performance (AUCs of 0.58 and 0.767). Finally, the combination of all three methods (radiomics, traditional radiological and clinical assessments) showed variable performance in four studies (AUCs 0.574 to 0.935). Based on this data, models lacking radiomic features appear to consistently underperform, while every model category that included radiomics reached higher performance peaks.

**Table 3:** Performance of the combined models in the included studies

Study	RF + TR models		RF + C models		TR + C models		RF + TR + C models	
	Testing set AUC (95% CI)	External validation set AUC (95% CI)	Testing set AUC (95% CI)	External v validation set AUC (95% CI)	Testing set AUC (95% CI)	External validation set AUC (95% CI)	Testing set AUC (95% CI)	External validation set AUC (95% CI)
Chen, Chao et al [14]								
Chen, Sihan et al [15]			0.912 (0.810–1.000)					
Cilla, Savino et al [16]								
Dong, Zheng et al [17]	0.852							
Ebrahimian, Shadi et al [18]								
Guan, Le et al [19]							0.935 (0.830–0.959)	
Gui, Chengzhi et al [20]								
Han, Ling et al [21]								
Han, Na et al [22]	0.82							
Hu, Weiming et al [23]			0.85 (0.761–0.915)	0.804 (0.718–0.874)				
Huang, Zhe et al [24]					0.58 (0.488–0.673)		0.916 (0.874–0.959)	
Huang, Zhe et al [25]							0.632 (0.598–0.665)	0.574 (0.499–0.650)/0.639 (0.528–0.750)
Lao, Qun et al [26]	0.993 (0.989–0.997)							
Le, Elizabeth P. V. et al [27]								
Le, Elizabeth P. V. et al [28]								
Li, Zhi-Li et al [29]	0.847							
Liu, M et al [30]			0.977 (0.899–0.999)	0.952 (0.884–0.987)				
Meddings, Zakaria et al [31]								
Shan, Dezhi et al [32]								
Shi, Jinglong et al [33]					0.767 (0.689–0.845)		0.832 (0.770–0.894)	
Song, Jiajia et al [34]								
Wang, Lingjie et al [35]								
Xia, Hai et al [36]			0.879					
Yu, Fan et al [37]	0.91							
Zhang, Lianlian et al [38]	0.87							
Zhang, Ranying et al [39]	0.986							
Zhang, Shuai et al [40]				0.811 (0.661–0.961)				
Zhang, Xun et al [41]	0.864 (0.72–1.00)							

*AUC* Area under the curve, *C* Clinical, *CI* Confidence interval, *RF* Radiomics features, *TR* Traditional radiological

CTA was the most utilized modality (*n* = 16), showing robust performance with AUC values ranging from 0.746 to 0.944. MRI-based radiomics (*n* = 9) achieved some of the highest recorded scores, peaking at an AUC of 0.984, with most models maintaining discriminatory power between 0.80 and 0.90. Conversely, US-based studies (*n* = 4) exhibited the widest range of performance, with AUCs varying from 0.606 to 0.922.

## Discussion

This systematic review summarized the existing literature on the radiomics-based assessment of carotid plaques and their performance in identifying high-risk lesions. By synthesizing evidence from 28 peer-reviewed studies involving 6,108 patients, radiomics emerged as a powerful, non-invasive tool capable of capturing the vulnerability of carotid plaques associated with embolic stroke risk. Notably, by transforming standard medical images into high-dimensional data, radiomics quantifies plaque heterogeneity through features invisible to the human eye.

Overall, the findings suggest that radiomics-based models provide superior discrimination of symptomatic lesions compared with traditional radiological and clinical assessments. Radiomics models outperformed other models in 83.3% of comparative analyses in testing (internal validation) sets. Furthermore, integrating radiomics with established clinical risk factors and traditional radiological markers consistently yields superior predictive performance compared to single-modality models, with combined signatures often achieving AUC values exceeding 0.90. This benefit stems from the complementary nature of the data: clinical data are useful for identifying independent risk factors; traditional radiological markers provide biological interpretability tied to known pathological processes; and radiomics provides incremental value by objectively quantifying sub-visual plaque heterogeneity and spatial complexity. The development of radiomics nomograms serves as a bridge to clinical practice, offering a user-friendly tool for personalized risk stratification that can significantly enhance the diagnostic accuracy and sensitivity of traditional visual assessments.

Radiomics-based models demonstrated strong capability in identifying high-risk carotid plaques across all imaging modalities. For future clinical translation, CTA-based radiomics represents a logical focus given this modality’s routine use in acute stroke management, lower cost, and higher potential for technical harmonization. Interestingly, research such as that by Chen et al [14] and Yu et al [37] demonstrates that incorporating PVAT features, which reflect perivascular inflammation, could provide incremental value in identifying high-risk plaques compared with plaque-only models. Pathophysiologically, PVAT is pertinent to carotid atheromatosis due to a complex relationship with the vessel wall, where vascular inflammation triggers paracrine signaling that induces transcriptional and structural changes in the surrounding fat [43]. These changes result in increased PVAT density, which can serve as a biomarker for atherosclerosis progression and high-risk plaque identification [44].

For these promising results to translate into routine clinical practice, several methodological hurdles must be addressed. Most of the included studies were retrospective and single-center studies, which inherently introduce selection bias and limit the generalizability of current models. There is a pronounced geographic concentration of research, with 24 of the 28 studies originating from China, potentially limiting the generalizability of these findings to global populations with different genetic or clinical profiles. Also, across the 28 studies included in this review, women are significantly underrepresented, with nearly three-quarters of participants being male. While this male predominance may reflect the higher epidemiological prevalence of carotid atherosclerotic disease in men, it is disproportionate to clinical stroke distributions. Large-artery atherosclerotic stroke reportedly accounts for 19.2% of ischemic strokes in men *versus* 12.7% in women, a ratio of approximately 1.5:1 (60% male and 40% female) [45]. The underrepresentation of females may introduce a population bias and limit the generalizability of the predictive models to this subgroup of patients. Moreover, many studies analyzed culprit arteries after a clinical event had already occurred, meaning the models may be characterizing the result of a stroke rather than predicting its onset.

The significant imaging protocol heterogeneity identified across the included studies represents a major technical hurdle that complicates the interpretation of radiomics data. Radiomic features are highly sensitive to the physical properties of the underlying image, such as resolution, signal-to-noise ratio, and voxel size. As detailed in Tables S5 and S6, the studies utilized hardware from different manufacturers, introducing an institutional bias where a model may inadvertently learn scanner-specific noise patterns or artifacts rather than true biological pathology. In MRI-based studies, although 3-T field strength was frequently used, the sequences were non-standardized, ranging from T1-weighted imaging to more advanced sequences. In CTA-based studies, tube voltages ranged from 80 to 150 kV, and slice thicknesses varied from 0.5 mm to 1.0 mm. Because Hounsfield units can shift based on tube voltage, features extracted from a 100 kV scan may not be comparable to those from a 150 kV scan. In future studies, researchers should adopt a standardized approach, including adherence to IBSI guidelines [42] and the use of robustness analyses to identify and exclude features sensitive to minor acquisition variations. Currently, only five studies explicitly confirmed compliance with the IBSI. This lack of standardization in feature-definition and extraction software (though PyRadiomics is widely used) makes it difficult to compare results across research groups.

Segmentation remains overwhelmingly manual (27/28 studies), which is time-consuming and prone to significant inter-observer variability. Another major problem is the lack of proper testing and validation of the models. Training, testing, and external validation datasets are essential in radiomics-based machine learning to prevent overfitting, assess generalizability, and ensure clinical reliability. Firstly, the training dataset teaches the model to learn patterns between radiomic features and outcomes, but radiomics’ high feature-to-patient ratio poses a substantial risk of overfitting, in which models learn spurious patterns rather than true biological relationships. Secondly, the testing dataset (internal validation) provides an initial assessment using patients from the same institution to detect overfitting, but this alone is insufficient because the data still share identical imaging protocols, scanner characteristics, and institutional practices. Thirdly, external validation using independent institutional data is essential to demonstrate true generalizability across real-world variability, including different equipment, acquisition protocols, and patient populations. As shown in Table 2, some studies do not report their models’ performance on validation datasets. For most, the models were cross-validated using five-fold cross-validation. While this method provides internal validation and helps detect overfitting within the development dataset, it does not replace the need for validation on completely independent data. In one study, the authors note that the number of cases across subgroups was insufficient to split the data into training and test sets [18]. Studies reporting only training performance should be considered, at best, preliminary and potentially invalid. In the 28 included studies, only 4 utilized an external validation cohort (TRIPOD Type 3). Without proper dataset partitioning across all three levels, models may learn imaging artifacts rather than pathology, yield unreliable performance estimates, and fail to translate clinically when applied to heterogeneous real-world data.

Regarding the RQS, the overall low scores highlight that radiomics is still in the exploratory phase. There is excellent documentation of imaging protocols, providing the transparency needed for reproducibility. However, the absence of phantom studies and multiple acquisition timepoints means that the stability of these RFs across different scanners or over time remains unproven. The lack of cost-effectiveness analysis and open science/data sharing prevents these models from being evaluated by healthcare policymakers or independently verified by other researchers.

Given this substantial methodological heterogeneity, conducting a meta-analysis of these studies would not be reliable. While the performance metrics (AUCs) are encouraging, the variation in how those metrics were achieved prevents a meaningful statistical synthesis. Firstly, the studies utilize a wide range of imaging tools. Because radiomic features are highly sensitive to the physical properties of the image (such as resolution, noise, and signal-to-noise ratio), pooling data from these different modalities would lead to invalid conclusions. Secondly, radiomics workflows and model construction methods vary significantly across the literature. Thirdly, the clinical parameters are highly heterogeneous, introducing population-specific variables that a meta-analysis cannot easily account for. In summary, the field of carotid radiomics is currently too fragmented and unstandardized to support a meta-analysis. Attempting to pool these results would likely introduce high statistical noise, obscuring the technology's true predictive value.

To overcome current methodological limitations, several technical and logistical improvements are essential to ensure the reliability and generalizability of radiomics-based risk assessments. There is a crucial need for large-scale, prospective longitudinal studies that follow patients over time to determine if radiomics can accurately predict future stroke events. The development of fully automated segmentation tools is essential to reduce human error, improve workflow efficiency, and ensure that radiomics can be implemented in a busy clinical environment. Future research must strictly adhere to IBSI guidelines and provide detailed descriptions of all pre-processing and extraction parameters to ensure reproducibility. Finally, more studies are needed to correlate radiomic features with histopathological findings, providing a biological basis for the mathematical patterns the models identify.

## Conclusion

In conclusion, radiomics provides a powerful, non-invasive approach to identifying vulnerable carotid plaques by extracting high-dimensional data on tissue heterogeneity that often eludes traditional visual assessment. This systematic review highlights that radiomics-based models consistently outperform conventional clinical and radiological metrics, such as the NASCET-defined degree of stenosis, particularly when integrated into combined predictive models. Despite these promising results, the transition to clinical practice is currently hindered by a lack of technical standardization and heavy reliance on manual segmentation. Future research must prioritize prospective multicenter trials and automated workflows to establish radiomics as a reliable and reproducible tool. Ultimately, the integration of radiomics into the diagnostic pipeline holds the potential to transform stroke prevention by providing a more precise and personalized assessment of carotid atherosclerotic disease.

## Supplementary information

ELECTRONIC SUPPLEMENTARY MATERIAL

## Data Availability

The data supporting the results reported in the article can be shared by the corresponding author upon request.

## Abbreviations

AUC: Area under the receiver operating characteristic curve
C: Clinical features or model
CTA: Computed tomography angiography
IBSI: Image Biomarker Standardization Initiative
MRI: Magnetic resonance imaging
NASCET: North American Symptomatic Carotid Endarterectomy Trial
PVAT: Perivascular adipose tissue
RF: Radiomics features
ROI: Region of interest
RQS: Radiomics Quality Score
TR: Traditional radiological features or model
TRIPOD: Transparent Reporting of a Multivariable Prediction Model for Individual Prognosis or Diagnosis
US: Ultrasonography
