# Supplementary material for: Radiomics performance to identify high-risk carotid artery plaques: a systematic review
Source: Eur Radiol Exp. 2026 Nov 3;10:170. doi: 10.1186/s41747-026-00843-7 (PMC13731619; doi:10.1186/s41747-026-00843-7)
Supplement: Supplementary file 1 [file 41747_2026_843_MOESM1_ESM.pdf]

Radiomics Performance to Identify High Risk Carotid Artery Plaques: A Systematic Review

ELECTRONIC SUPPLEMENTARY MATERIAL

Search strategy:

This search strategy was developed and executed with the help of two librarians from the University of Sherbrooke (Mireille Léger-Rousseau and Jacinthe Stornello).

1) PubMed:

("Radiomics"[Mesh] OR radiomic OR "texture analysis" OR "artificial intelligence"[Mesh] OR "Artificial Intelligence" OR AI OR "Machine Learning"[Mesh] OR "machine learning" OR algorithm OR "Deep Learning"[Mesh] OR "deep learning")

AND

("Carotid Stenosis"[Mesh] OR "Carotid Artery Diseases"[Mesh] OR "Atherosclerotic Plaque"[Mesh] OR "carotid stenosis" OR "carotid artery stenosis" OR "carotid atherosclerosis" OR "vulnerable plaque" OR "unstable plaque" OR "high-risk plaque" OR "high-risk stenosis")

2) Embase:

('radiomics'/exp OR radiomic*:ab,ti OR "texture analysis":ab,ti OR 'artificial intelligence'/exp OR "artificial intelligence":ab,ti OR AI:ab,ti OR 'machine learning'/exp OR "machine learning":ab,ti OR algorithm*:ab,ti OR 'deep learning'/exp OR "deep learning":ab,ti)

AND

('carotid stenosis'/exp OR "carotid stenosis":ab,ti OR "carotid artery stenosis":ab,ti OR 'carotid artery disease'/exp OR "carotid artery disease":ab,ti OR 'atherosclerotic plaque'/exp OR "carotid atherosclerosis":ab,ti OR "vulnerable plaque":ab,ti OR "unstable plaque":ab,ti OR "high-risk plaque":ab,ti OR "high-risk stenosis":ab,ti)

3) Scopus:

(TITLE-ABS-KEY(radiomic* OR "texture analysis" OR "artificial intelligence" OR AI OR "machine learning" OR algorithm* OR "deep learning"))

AND

(TITLE-ABS-KEY("carotid stenosis" OR "carotid artery stenosis" OR "carotid artery disease*" OR "carotid atherosclerosis" OR "atherosclerotic plaque" OR "vulnerable plaque" OR "unstable plaque" OR "high-risk plaque" OR "high-risk stenosis"))

(TITLE-ABS-KEY(radiomic OR texture analysis OR artificial intelligence OR AI OR machine learning OR algorithm OR deep learning))

AND

(TITLE-ABS-KEY(carotid stenosis OR carotid artery stenosis OR carotid artery disease OR carotid atherosclerosis OR atherosclerotic plaque OR vulnerable plaque OR unstable plaque OR high-risk plaque OR high-risk stenosis))

4) Cochrane Library:

(radiomic* OR "texture analysis" OR "artificial intelligence" OR AI OR "machine learning" OR algorithm* OR "deep learning")

AND

("carotid stenosis" OR "carotid artery stenosis" OR "carotid artery disease*" OR "carotid atherosclerosis" OR "atherosclerotic plaque" OR "vulnerable plaque" OR "unstable plaque" OR "high-risk plaque" OR "high-risk stenosis")

5) Google Scholar:

("radiomics" OR "texture analysis")

AND

("carotid stenosis" OR "carotid artery stenosis" OR "carotid artery disease" OR "carotid atherosclerosis")

6) Manual search from previously published reviews

PRISMA 2020 checklist

Section and Topic	Item #	Checklist item	Location where item is reported
**TITLE**			
Title	1	Identify the report as a systematic review.	Page 1
**ABSTRACT**			
Abstract	2	See the PRISMA 2020 for Abstracts checklist.	Pages 1-2
**INTRODUCTION**			
Rationale	3	Describe the rationale for the review in the context of existing knowledge.	Pages 4-6
Objectives	4	Provide an explicit statement of the objective(s) or question(s) the review addresses.	Page 6
**METHODS**			
Eligibility criteria	5	Specify the inclusion and exclusion criteria for the review and how studies were grouped for the syntheses.	Pages 7-8
Information sources	6	Specify all databases, registers, websites, organisations, reference lists and other sources searched or consulted to identify studies. Specify the date when each source was last searched or consulted.	Page 7 & 9
Search strategy	7	Present the full search strategies for all databases, registers and websites, including any filters and limits used.	Page 7
Selection process	8	Specify the methods used to decide whether a study met the inclusion criteria of the review, including how many reviewers screened each record and each report retrieved, whether they worked independently, and if applicable, details of automation tools used in the process.	Pages 7-9
Data collection process	9	Specify the methods used to collect data from reports, including how many reviewers collected data from each report, whether they worked independently, any processes for obtaining or confirming data from study investigators, and if applicable, details of automation tools used in the process.	Pages 8-9
Data items	10a	List and define all outcomes for which data were sought. Specify whether all results that were compatible with each outcome domain in each study were sought (e.g. for all measures, time points, analyses), and if not, the methods used to decide which results to collect.	Pages 8-9 Tables 1-3
	10b	List and define all other variables for which data were sought (e.g. participant and intervention characteristics, funding sources). Describe any assumptions made about any missing or unclear information.	Pages 8-9 Tables 1-3
Study risk of bias assessment	11	Specify the methods used to assess risk of bias in the included studies, including details of the tool(s) used, how many reviewers assessed each study and whether they worked independently, and if applicable, details of automation tools used in the process.	-
Effect measures	12	Specify for each outcome the effect measure(s) (e.g. risk ratio, mean difference) used in the synthesis or presentation of results.	Page 9

Synthesis methods	13a	Describe the processes used to decide which studies were eligible for each synthesis (e.g. tabulating the study intervention characteristics and comparing against the planned groups for each synthesis (item #5)).	-
	13b	Describe any methods required to prepare the data for presentation or synthesis, such as handling of missing summary statistics, or data conversions.	-
	13c	Describe any methods used to tabulate or visually display results of individual studies and syntheses.	Tables 1-3<br>Tables S1-3
	13d	Describe any methods used to synthesize results and provide a rationale for the choice(s). If meta-analysis was performed, describe the model(s), method(s) to identify the presence and extent of statistical heterogeneity, and software package(s) used.	-
	13e	Describe any methods used to explore possible causes of heterogeneity among study results (e.g. subgroup analysis, meta-regression).	-
	13f	Describe any sensitivity analyses conducted to assess robustness of the synthesized results.	-
Reporting bias assessment	14	Describe any methods used to assess risk of bias due to missing results in a synthesis (arising from reporting biases).	-
Certainty assessment	15	Describe any methods used to assess certainty (or confidence) in the body of evidence for an outcome.	-
**RESULTS**			
Study selection	16a	Describe the results of the search and selection process, from the number of records identified in the search to the number of studies included in the review, ideally using a flow diagram.	Pages 9-10<br>Figure 2
	16b	Cite studies that might appear to meet the inclusion criteria, but which were excluded, and explain why they were excluded.	Pages 9-10
Study characteristics	17	Cite each included study and present its characteristics.	Page 10
Risk of bias in studies	18	Present assessments of risk of bias for each included study.	-
Results of individual studies	19	For all outcomes, present, for each study: (a) summary statistics for each group (where appropriate) and (b) an effect estimate and its precision (e.g. confidence/credible interval), ideally using structured tables or plots.	Tables 1-3
Results of syntheses	20a	For each synthesis, briefly summarise the characteristics and risk of bias among contributing studies.	P. 10-14
	20b	Present results of all statistical syntheses conducted. If meta-analysis was done, present for each the summary estimate and its precision (e.g. confidence/credible interval) and measures of statistical heterogeneity. If comparing groups, describe the direction of the effect.	P. 10-14
	20c	Present results of all investigations of possible causes of heterogeneity among study results.	-
	20d	Present results of all sensitivity analyses conducted to assess the robustness of the synthesized results.	-
Reporting biases	21	Present assessments of risk of bias due to missing results (arising from reporting biases) for each synthesis assessed.	-

Certainty of evidence	22	Present assessments of certainty (or confidence) in the body of evidence for each outcome assessed.	-
**DISCUSSION**			
Discussion	23a	Provide a general interpretation of the results in the context of other evidence.	P. 14-15
	23b	Discuss any limitations of the evidence included in the review.	P. 15-17
	23c	Discuss any limitations of the review processes used.	Page 17
	23d	Discuss implications of the results for practice, policy, and future research.	Page 18
**OTHER INFORMATION**			
Registration and protocol	24a	Provide registration information for the review, including register name and registration number, or state that the review was not registered.	Page 7
	24b	Indicate where the review protocol can be accessed, or state that a protocol was not prepared.	Page 7
	24c	Describe and explain any amendments to information provided at registration or in the protocol.	Page 7
Support	25	Describe sources of financial or non-financial support for the review, and the role of the funders or sponsors in the review.	-
Competing interests	26	Declare any competing interests of review authors.	-
Availability of data, code and other materials	27	Report which of the following are publicly available and where they can be found: template data collection forms; data extracted from included studies; data used for all analyses; analytic code; any other materials used in the review.	-

Table S1. Main purpose of the included studies

1) Chen, Chao et al. 2023	To develop and test radiomics models of carotid plaques and perivascular adipose tissue to distinguish symptomatic from asymptomatic plaques and compare the diagnostic value between radiomics models and traditional CTA model.
2) Chen, Sihan et al. 2022	To construct a radiomics-based MRI sequence high-resolution MRI, combined with clinical high-risk factors for non-invasive differentiation of the plaque of symptomatic patients from asymptomatic patients.
3) Cilla, Savino et al. 2022	To develop a CTA-based radiomics analysis and machine learning model for carotid plaques to discriminate vulnerable from no vulnerable plaques.
4) Dong, Zheng et al. 2022	To evaluate the diagnostic performance of radiomics-based machine learning assessment of carotid CTA data to identify symptomatic patients with carotid artery atherosclerosis.
5) Ebrahimian, Shadi et al. 2022	To compare spectral segmentation, spectral radiomic, and single-energy radiomic features in the assessment of internal carotid artery/common carotid artery stenosis and prediction of surgical outcomes.
6) Guan, Le et al. 2024	To develop and validate a clinical-radiomics nomogram for predicting early ischemic stroke risk in patients who sustain a transient ischemic attack.
7) Gui, Chengzhi et al. 2023	To investigate the classification of symptomatic plaques by evaluating the models generated via two different approaches, a radiomics-based machine learning approach, and an end-to-end learning approach which utilized deep learning techniques.
8) Han, Ling et al. 2024	To compare the MRI and CT imaging characteristics of carotid plaques, assess the machine learning-based imaging prediction model's capacity to identify high-risk and severe asymptomatic carotid stenosis patients.
9) Han, Na et al. 2024	To accurately predict the risk of ischemic stroke, we established a radiomics model of carotid atherosclerotic plaque-based high-resolution vessel wall magnetic resonance imaging and combined it with clinical indicators.
10) Hu, Weiming et al. 2025	To create a radiomics nomogram using dual-energy computed tomography virtual monoenergetic images to accurately identify symptomatic carotid plaques.
11) Huang, Zhe et al. 2022	To build a nomogram using ultrasonography-based radiomics and clinical features for identification of symptomatic carotid plaques.
12) Huang, Zhe et al. 2024	To assess whether a radiomics-based nomogram correlates with a higher risk of future cerebro-cardiovascular events in patients with asymptomatic carotid plaques.
13) Lao, Qun et al. 2024	To develop a radiomics model to assess carotid artery plaque vulnerability using computed tomography angiography images.

14) Le, Elizabeth P. V. et al. 2021	To understand whether radiomic features extracted from standard clinical CT scans were robust and reliable and whether they could provide additional prognostic information to help identify higher-risk culprit arteries from lower-risk non-culprit carotid arteries.
15) Le, Elizabeth P. V. et al. 2024	To assess radiomics and deep learning methods in identifying symptomatic carotid artery disease from carotid CTA images, and to compare the performance of these novel methods to the conventional calcium score.
16) Li, Zhi-li et al. 2025	To explore the value of a model constructed by integrating CTA-based carotid plaque radiomic features, clinical risk factors, and plaque imaging characteristics for prognosticating the risk of ischemic stroke.
17) Liu, M et al. 2023	To develop and validate a radiomics nomogram for identifying high-risk carotid plaques on CTA.
18) Meddings, Zakaria et al. 2024	To assess how radiomic features may be combined with plaque morphological and compositional features identified by multi-contrast MRI to improve upon conventional risk assessment models in determining culprit carotid artery lesions.
19) Shan, Dezhi et al. 2023	To identify radiomic features associated with the neovascularization of carotid atherosclerotic plaque and construct a prediction model for plaque vulnerability based on radiomic features.
20) Shi, Jinglong et al. 2023	To develop and validate a combined model incorporating conventional clinical and imaging characteristics and radiomics signatures based on head and neck CTA to assess plaque vulnerability.
21) Song, Jiajia et al. 2025	Utilizing artificial intelligence technology for the segmentation of plaques on ultrasound images to evaluate the stability of carotid artery plaques and analyze its diagnostic accuracy in differentiating vulnerable plaques from stable ones.
22) Wang, Lingjie et al. 2024	To explore the effectiveness of a deep learning-based three-dimensional super-resolution CTA radiomic model for improved identification of symptomatic carotid atherosclerotic plaques.
23) Xia, Hai et al. 2023	To establish a radiomics-based machine learning model that predicts the risk of transient ischemic attack in patients with mild carotid stenosis (30–50% NASCET) using extracted computed tomography radiomics features and clinical information.
24) Yu, Fan et al. 2025	To develop an ensemble model based on carotid plaque and perivascular adipose tissue MRI radiomics for identifying highly inflammatory plaques.
25) Zhang, Lianlian et al. 2022	To determine whether texture analysis based on a vascular ultrasound can be applied to identify vulnerable plaques.
26) Zhang, Ranying et al. 2021	To build a high-risk plaque MRI-based model using radiomics features and machine learning for differentiating symptomatic from asymptomatic carotid plaques.
27) Zhang, Shuai et al. 2022	To develop and validate a CT-based radiomics nomogram incorporating clinical factors and radiomics signature for the detection of intra-plaque hemorrhage in carotid arteries.
28) Zhang, Xun et al. 2023	To develop a 3D carotid plaque radiomics model based on high-resolution MRI to quantitatively identify vulnerable plaques.

Legend: CTA: computed tomography angiography, MRI: magnetic resonance imaging, NASCET: North American Symptomatic Carotid Endarterectomy Trial.

Table S2. Image segmentation and feature extraction methodologies in the included studies

Study	Type of features (RF, TR, C)	Imaging modality	ROI	Single or Multi slices	Segmentation method	IBSI compliance mentioned	N of RF in the final model	External validation	Extraction software
Chen, Chao et al., 2023	RF, TR (RF plaques, RF PVAT, combinations)	CTA	Plaques + PVAT at level of maximum plaque area	Single slice	Manual (plaques) and semi-automated (attenuation threshold)	No	3	No	3D Slicer (extension not specified)
Chen, Sihan et al., 2022	RF, C, RF+C	HR-MRI	Max plaque	Single slice	Manual	Yes	16	No	Pyradiomics
Cilla, Savino et al., 2022	RF	CTA	Entire plaques	Multi slices	Manual	Yes	2	No	Moddicom
Dong, Zheng et al., 2022	RF, TR, RF+TR	CTA	Plaque boundaries at largest plaque area	Single slice	Manual	No	20	No	Deepwise multimodal research platform
Ebrahimian, Shadi et al., 2022	RF, TR	CTA (dual energy)	Lumen and vessel wall, Max stenosis and entire length of stenosis	Single and Multi slices	Manual	No	4	No	Pyradiomics
Guan, Le et al., 2024	RF, TR, C, RF+TR+C	HR-MRI	Entire plaques	Multi slices	Manual	No	7	No	Artificial Intelligent Kit
Gui, Chengzhi et al., 2023	RF	HR-MRI	Entire plaques	Multi slices	Manual	Yes	?	No	Pyradiomics
Han, Ling et al. 2024	RF	CTA and MRI	Contour of the plaques	Multi slices	Manual	No	4	No	Pyradiomics
Han, Na et al., 2024	RF, TR, RF+TR	HR-VWMRI	Entire plaques	Multi slices	Manual	No	10 and 8	No	Pyradiomics
Hu, Weiming et al., 2025	RF, C, RF+C	CTA (dual energy)	Plaques	Multi slices	Manual	No	13, 12, 10, 18, 14, 13, 21	Yes (1 hospital)	Radcloud platform
Huang, Zhe et al., 2022	RF, TR+C, RF+TR+C	US	Sections containing the largest plaque area	Single slice	Manual	No	6	No	R software
Huang, Zhe et al., 2024	RF, TR, C, RF+TR+C	US	Image with largest plaque area	Single slice	Manual	No	18	Yes (2 hospitals)	Pyradiomics
Lao, Qun et al., 2024	RF, RF+TR	CTA	Entire plaques	Multi slices	Manual	No	13	No	Pyradiomics
Le, Elizabeth P. V. et al., 2021	RF (single vs multi slices)	CTA	Plaques (carotid artery adventitia (outer wall))	Single and Multi slices	Manual	No	10	No	Pyradiomics

Le, Elizabeth P. V. et al., 2024	RF	CTA	Plaques (outer vessel wall)	Single and Multi slices	Manual	No	93	No	Pyradiomics
Li, Zhi-Li et al., 2025	RF, TR, RF+TR	CTA	Plaque on the most stenotic slice	Single slice	Manual	No	9	No	Pyradiomics
Liu, M et al., 2023	RF, C, RF+C	CTA	Plaques (cross-sectional area)	Multi slices	Manual	No	24	Yes (1hospital)	Radcloud platform
Meddings, Zakaria et al., 2024	RF, TR, RF+TR	MRI	Pixels from slices with atherosclerotic disease	Multi slices	Manual	No	107	No	Pyradiomics
Shan, Dezhi et al., 2023	RF, C	CTA	Plaques	Multi slices	Manual	No	10	No	Pyradiomics
Shi, Jinglong et al., 2023	RF, TR+C, RF+TR+C	CTA	Plaques	Multi slices	Manual	No	6	No	Deepwise multimodal research platform
Song, Jiajia et al., 2025	RF, C	US	Plaques	2D segmentation	Automated	No	43	No	Image Analyzer 2.0
Wang, Lingjie et al., 2024	RF, TR	CTA (dual energy)	Plaques	Multi slices	Manual	Yes	5 and 18	No	Pyradiomics
Xia, Hai et al., 2023	RF, C, RF+C	CTA	Plaques	Multi slices	Manual	No	9	No	Pyradiomics
Yu, Fan et al. 2025	RF (plaque and PVAT), TR, RF+TR	MRI (and PET)	Carotid artery wall in which the plaque is (and PVAT)	Multi slices	Manual	No	17	No	Pyradiomics
Zhang, Lianlian et al. 2022	RF, TR, RF+TR	US	Maximum area of the plaque on longitudinal section	Single slice	Manual	No	7	No	MaZda
Zhang, Ranying et al., 2021	RF, TR, RF+TR	MRI	Plaques on slice with largest plaque	Single slice	Manual	No	33	No	Pyradiomics
Zhang, Shuai et al., 2022	RF, C, RF+C	CTA (and MRI for IPH)	Within the border of the plaque	Multi slices	Manual	No	8	Yes (1 hospital)	Huiying Medical Technology Co., Ltd
Zhang, Xun et al., 2023	RF, TR, RF+TR	HR-MRI	Entire plaques	Multi slices	Manual	Yes	13	No	Pyradiomics

Legend: C: clinical, CTA: computed tomography angiography, HR-MRI: high-resolution magnetic resonance imaging, HR-VWMRI: high-resolution vessel wall magnetic resonance imaging, IBSI: Image Biomarker Standardization Initiative, IPH: intraplaque hemorrhage, MRI: magnetic resonance imaging, N: number, PET: positron emission tomography, PVAT: perivascular adipose tissue, RF: radiomics features, TR: traditional radiological, US: ultrasonography.

Table S3. Performance of the best predictive models in the included studies (training sets)

Study	Training set		
	RF model<br>AUC (95% CI)	TR model<br>AUC (95% CI)	C model<br>AUC (95% CI)
Chen, Chao et al., 2023	0.766<br>(0.666-0.865)	0.624<br>(0.506-0.743)	
Chen, Sihan et al., 2022	0.834<br>(0.753-0.942)		0.852<br>(0.771-0.943)
Cilla, Savino et al., 2022	0.987		
Dong, Zheng et al., 2022			
Ebrahimian, Shadi et al., 2022	0.76<br>(0.66-0.85)	0.63<br>(0.45-0.73)	
Guan, Le et al., 2024	0.737<br>(0.715-0.878)	0.704<br>(0.535-0.854)	0.752<br>(0.666-0.862)
Gui, Chengzhi et al., 2023	0.8611		
Han, Ling et al. 2024	0.872<br>(0.813-0.931)		
Han, Na et al., 2024	0.82	0.79	
Hu, Weiming et al., 2025	0.873<br>(0.820-0.914)		0.794<br>(0.734-0.847)
Huang, Zhe et al., 2022	0.931<br>(0.903-0.959)		
Huang, Zhe et al., 2024	0.678<br>(0.642-0.714)	0.629<br>(0.591-0.668)	0.612<br>(0.575-0.649)
Lao, Qun et al., 2024	0.972<br>(0.961-0.983)	0.892<br>(0.830-0.954)	
Le, Elizabeth P. V. et al., 2021	0.67		
Le, Elizabeth P. V. et al., 2024	0.96		
Li, Zhi-Li et al., 2025	0.766	0.766	
Liu, M et al., 2023	0.976<br>(0.934-0.995)		0.845<br>(0.773-0.902)
Meddings, Zakaria et al., 2024	0.805	0.689	
Shan, Dezhi et al., 2023	1.00		0.9
Shi, Jinglong et al., 2023	0.84<br>(0.781-0.898)		
Song, Jiajia et al., 2025	0.894<br>(0.807-0.981)		0.7
Wang, Lingjie et al., 2024	0.907<br>(0.857-0.957)	0.776<br>(0.709-0.842)	
Xia, Hai et al., 2023	0.982		0.981
Yu, Fan et al. 2025	0.84	0.68	
Zhang, Lianlian et al. 2022	0.8	0.77	
Zhang, Ranying et al., 2021	0.988	0.825	
Zhang, Shuai et al., 2022	0.717<br>(0.620-0.814)		0.631<br>(0.524-0.738)
Zhang, Xun et al., 2023	0.915<br>(0.85-0.98)	0.816<br>(0.70-0.93)	

Legend: AUC (area under the curve), C: clinical, CI: confidence interval, RF: radiomics features, TR: traditional radiological.

The data included in Table S3 does not represent true performance of the models. Overfitting risk is high, and data should be interpreted with caution.

Table S4. Performance of the different radiomics models in the included studies

**Study**	**Radiomics model - Testing set**<br>**AUC (95% CI)**
Chen, Chao et al., 2023	SVM: 0.759 (0.606-0.911)
Chen, Sihan et al., 2022	LR: 0.818 (0.674-0.962)
Cilla, Savino et al., 2022	
Dong, Zheng et al., 2022	LR: 0.858 (0.782-0.933)<br>XGBoost: data not provided<br>SVM: data not provided
Ebrahimian, Shadi et al., 2022	
Guan, Le et al., 2024	LR + RF: 0.899 (0.706-0.936)
Gui, Chengzhi et al., 2023	
Han, Ling et al. 2024	LR: 0.867 (0.776-0.959)<br>NB: 0.855 (0.755-0.954)<br>KNN: 0.799 (0.691-0.907)<br>SVM: 0.761 (0.623-0.870)<br>RF: 0.710 (0.558-0.802)<br>DT: 0.697 (0.568-0.827)
Han, Na et al., 2024	LR: 0.74
Hu, Weiming et al., 2025	SVM: 0.824 (0.731-0.895)
Huang, Zhe et al., 2022	LR: 0.922 (0.878-0.966)
Huang, Zhe et al., 2024	Multivariate Cox proportional hazard regression: 0.606 (0.573-0.638)
Lao, Qun et al., 2024	LR: 0.942 (0.918-0.999)
Le, Elizabeth P. V. et al., 2021	
Le, Elizabeth P. V. et al., 2024	
Li, Zhi-Li et al., 2025	LR: 0.801
Liu, M et al., 2023	LR: 0.944 (0.850-0.987)
Meddings, Zakaria et al., 2024	
Shan, Dezhi et al., 2023	RF: 0.933 (0.880-0.985)<br>LR: 0.731 (0.651-0.811)<br>MLP: 0.729 (0.637-0.820)<br>XGBoost: 0.727 (0.604-0.849)<br>SVM: 0.717 (0.713-0.721)<br>AdaBoost: 0.644 (0.471-0.817)<br>LGBM: 0.635 (0.581-0.688)
Shi, Jinglong et al., 2023	LR: 0.797 (0.731-0.863)
Song, Jiajia et al., 2025	SVM: 0.827 (0.716-0.938)
Wang, Lingjie et al., 2024	SVM: 0.82 (0.756-0.884)
Xia, Hai et al., 2023	RF: 0.746<br>XGBoost: 0.711<br>KNN: 0.583<br>LR: 0.560<br>SVM: 0.500
Yu, Fan et al. 2025	Multi-layer cascade reduction dimension algorithm : 0.85
Zhang, Lianlian et al. 2022	LR: 0.83
Zhang, Ranying et al., 2021	LR: 0.984
Zhang, Shuai et al., 2022	
Zhang, Xun et al., 2023	LR: 0.835 (0.68-0.99)

Legend: AdaBoost: adaptive boosting, AUC: area under the curve, C: clinical, CI: confidence interval, DT: decision tree, KNN: K-nearest neighbors, LGBM: light gradient boosting machine, LR: logistic regression, MLP: multi-layer perception, NB: naive Bayes, RF: random forest, SVM: support vector machine, TR: traditional radiological, XGBoost: extreme gradient boosting.

Table S5. CTA-based studies acquisition protocol and imaging information

Study	Acquisition protocol	Scanner type	Scan duration	Tube voltage	Tube current	Slice thickness	Slice spacing
Chen, Chao et al., 2023	Arch to skull apex	SOMATOM		100 kV	300 mA	0,6 mm	0,5 mm
Chen, Sihan et al., 2022							
Cilla, Savino et al., 2022		Brilliance 128				0,9 mm	
Dong, Zheng et al., 2022		Somaton Definition Flash		120 kV	300 mA	0,75 mm	0,5 mm
Ebrahimian, Shadi et al., 2022	Dual-energy scan mode	Siemens Definition Flash		80 and 140 kV	320 mA	1 mm	0,5 mm
Guan, Le et al., 2024							
Gui, Chengzhi et al., 2023							
Han, Ling et al. 2024	Arch to skull apex	Philips Brilliance					
Han, Na et al., 2024							
Hu, Weiming et al., 2025	DECT head and neck	SOMATOM Force		80 and 150 kV	Automatic modulation	0,5 mm	0,5 mm
Huang, Zhe et al., 2022							
Huang, Zhe et al., 2024							
Lao, Qun et al., 2024	Neck CTA	GE Healthcare		100 kV	120 mA	1 mm	1 mm
Le, Elizabeth P. V. et al., 2021	Arch to circle of Willis	GE Discovery combined 690 PET-CT		120 kV	200 mA	0,625 mm	0,4 mm
Le, Elizabeth P. V. et al., 2024	Arch to circle of Willis	GE Discovery combined 690 PET-CT				0,625 mm	0,4 mm
Li, Zhi-Li et al., 2025	Arch to skull apex	Philips 256-slice spiral CT scanner		120 kV	Automatic modulation	0,9 mm	0,45 mm
Liu, M et al., 2023	Arch to skull apex	SOMATOM Force		110 kV		0,5 mm	0,5 mm
Meddings, Zakaria et al., 2024							
Shan, Dezhi et al., 2023	Carotid CTA	AquilionONE		80 kV	100 mA	0,5 mm	
Shi, Jinglong et al., 2023	Arch to skull vertex	Brilliance iCT		120 kV	250 mA	0,9 mm	0,45 mm
Song, Jiajia et al., 2025							
Wang, Lingjie et al., 2024	Head and neck CTA	SOMATOM Force		100 and 150 kV		0,75 mm	0,5 mm
Xia, Hai et al., 2023	Carotid CTA	Somaton Definition Flash DSCT		120 kV		0,625 mm	0,625 mm
Yu, Fan et al. 2025							
Zhang, Lianlian et al. 2022							
Zhang, Ranying et al., 2021							
Zhang, Shuai et al., 2022	Arch to skull apex	SOMATOM Force		100 kV		0,5 mm	0,5 mm
Zhang, Xun et al., 2023							

Legend: CTA: computed tomography angiography, DECT: dual-energy computed tomography, kV: kilovolt, mA: milliampere, mm: millimeter

Table S6. MRI-based studies acquisition protocol and imaging information

Study	Acquisition protocol	Scanner type	Duration	RF coil	Scanning sequence	Magnetic field strength	Slice thickness	Slice spacing
Chen, Chao et al., 2023								
Chen, Sihan et al., 2022		MR750 system (GE)		8-channel carotid coil	T1, T2, PD, 3D TOF	3 T	2 mm	
Cilla, Savino et al., 2022								
Dong, Zheng et al., 2022								
Ebrahimian, Shadi et al., 2022								
Guan, Le et al., 2024	HR-MRI extracranial carotid arteries	Magnetom Verio	29 min 37 sec		T1W-FS, T2W-FS, 3D-TOF	3 T	2 mm	
Gui, Chengzhi et al., 2023		MAGNETOM Prisma		64-channel head/neck coil	SPACE, DWI, DSC-PWI	3 T	1/5 mm	
Han, Ling et al. 2024		Magnetom Verio				3 T		
Han, Na et al., 2024	Carotid and head MRI sequences		29 min	20-channel head/neck coil	TOF, T1, T2, T1-CE, DWI, FLAIR	3 T	Table 1	
Hu, Weiming et al., 2025								
Huang, Zhe et al., 2022								
Huang, Zhe et al., 2024								
Lao, Qun et al., 2024								
Le, Elizabeth P. V. et al., 2021								
Le, Elizabeth P. V. et al., 2024								
Li, Zhi-Li et al., 2025								
Liu, M et al., 2023								
Meddings, Zakaria et al., 2024	Carotid MRI / vessel wall imaging	Philips Achieva TX		8-channel carotid coil	3D TOF, T1, QIR, T2, MDIR, MPRAGE			
Shan, Dezhi et al., 2023								
Shi, Jinglong et al., 2023	Brain MRI (*not for carotid arteries)	Discovery 750		20-channel head coil	T1, T2, FLAIR, DWI	3 T	6 mm (DWI)	1,2 mm (DWI)
Song, Jiajia et al., 2025								
Wang, Lingjie et al., 2024								
Xia, Hai et al., 2023								
Yu, Fan et al. 2025			20 min 16 sec		3D-FS-T1 FSE, 3D-FS-T2 FSE, 3D-FS-T1 FSE CE			

Zhang, Lianlian et al. 2022	HR-MRI	GE Signa HDXT			3D TOF MRA, black-blood sequence (T1WI, T2WI, PDWI)	3 T		
Zhang, Ranying et al., 2021	Carotid MR pulse sequences	MAGNETOM Verio	22 min	16-channel head coil, 8-channel carotid coil	3D TOF, T1W TSE, T2W TSE, DCE 3D VIBE, post-contrast T1W TSE	3 T		
Zhang, Shuai et al., 2022	HR-MRI	Prisma, Siemens		64-channel head/neck coil	Details in supp material, Table S1	3 T		
Zhang, Xun et al., 2023	HR-MRI	Magnetom Verio		64-channel coil	3D T1-SPACE +/-CE	3 T	2 mm (DWI)	

Legend: 3D: 3-dimensional, CE: contrast-enhanced, DCE: dynamic contrast-enhanced, DSC-PWI: dynamic susceptibility contrast perfusion-weighted imaging, DWI: diffusion-weighted imaging, FLAIR: fluid-attenuated inversion recovery, FS: fat saturation, FSE: fast spin echo, HR-MRI: high resolution MRI, MDIR: multi-slice double inversion recovery, min: minutes, mm: millimeter, MPRAGE: magnetization-prepared rapid gradient echo, MRA: magnetic resonance angiography, MRI: magnetic resonance imaging, PD: proton density, QIR: quadruple inversion-recovery, sec: seconds, T: Tesla, T1: T1-weighted, T2: T2-weighted, TOF: time-of-flight, TSE: turbo spin echo

Table S7. Transparent Reporting of a multivariable prediction model for Individual Prognosis Or Diagnosis (TRIPOD) classification of the included studies

TRIPOD type	Analysis description	Included studies
Type 1a	Development of a prediction model where predictive performance is then directly evaluated using exactly the same data (apparent performance).	
Type 1b	Development of a prediction model using the entire data set, but then using resampling (e.g., bootstrapping or cross-validation) techniques to evaluate the performance and optimism of the developed model. Resampling techniques, generally referred to as "internal validation", are recommended as a prerequisite for prediction model development, particularly if data are limited.	Cilla (2022)<br>Dong (2022)<br>Ebrahimian (2022)<br>Gui (2023)<br>Le (2021)<br>Le (2024)<br>Meddings (2024)<br>Wang (2024)
Type 2a	The data are randomly split into 2 groups: one to develop the prediction model, and one to evaluate its predictive performance. This design is generally not recommended or better than type 1b, particularly in case of limited data, because it leads to lack of power during model development and validation.	Chen S. (2022)<br>Chen C. (2023)<br>Han L. (2024)<br>Han N. (2024)<br>Huang (2022)<br>Lao (2024)<br>Li (2025)<br>Shan (2023)<br>Shi (2023)<br>Song (2025)<br>Xia (2023)<br>Yu (2025)<br>Zhang R. (2021)<br>Zhang X. (2023)<br>Zhang L. (2022)
Type 2b	The data are nonrandomly split (e.g., by location or time) into 2 groups: one to develop the prediction model and one to evaluate its predictive performance. Type 2b is a stronger design for evaluating model performance than type 2a, because allows for nonrandom variation between the 2 data sets.	Guan (2024)
Type 3	Development of a prediction model using 1 data set and an evaluation of its performance on separate data (e.g., from a different study).	Hu (2025)<br>Huang (2024)<br>Liu (2023)<br>Zhang S. (2022)

Type 4	Evaluation of the predictive performance of an existing (published) prediction model on separate data.	

Table S8. Radiomics Quality Score of the included studies

Study	1	2	3	4	5	6	7	8	9	10	11	12	13	14	15	16	RQS
Chen, Chao et al., 2023	Green	Red	Red	Red	Green	Green	Green	Red	Green	Red	Red	Orange	Red	Green	Red	Red	11
Chen, Sihan et al., 2022	Green	Green	Red	Red	Green	Green	Green	Green	Green	Green	Red	Orange	Red	Green	Red	Red	14
Cilla, Savino et al., 2022	Green	Green	Red	Red	Green	Red	Green	Red	Green	Red	Red	Red	Green	Green	Red	Red	11
Dong, Zheng et al., 2022	Green	Green	Red	Red	Green	Green	Green	Red	Green	Red	Red	Orange	Red	Green	Red	Red	12
Ebrahimian, Shadi et al., 2022	Green	Green	Red	Red	Green	Red	Green	Red	Green	Red	Red	Red	Red	Green	Red	Red	9
Guan, Le et al., 2024	Green	Green	Red	Red	Green	Green	Green	Red	Green	Green	Red	Orange	Red	Green	Red	Red	13
Gui, Chengzhi et al., 2023	Green	Green	Red	Red	Green	Red	Green	Red	Green	Red	Red	Orange	Red	Green	Red	Red	11
Han, Ling et al. 2024	Green	Green	Red	Red	Green	Red	Green	Red	Green	Green	Red	Orange	Red	Green	Red	Red	12
Han, Na et al., 2024	Green	Green	Red	Red	Green	Green	Green	Green	Green	Red	Red	Orange	Red	Green	Red	Red	13
Hu, Weiming et al., 2025	Green	Green	Red	Red	Green	Green	Green	Green	Green	Green	Red	Green	Red	Green	Red	Red	15
Huang, Zhe et al., 2022	Green	Green	Red	Red	Green	Green	Green	Green	Green	Red	Red	Orange	Red	Green	Red	Red	13
Huang, Zhe et al., 2024	Green	Green	Red	Red	Green	Green	Green	Green	Green	Green	Red	Green	Red	Green	Red	Red	16
Lao, Qun et al., 2024	Green	Red	Red	Red	Green	Green	Green	Green	Green	Green	Red	Orange	Green	Green	Red	Red	15
Le, Elizabeth P. V. et al., 2021	Green	Green	Red	Red	Green	Green	Green	Red	Green	Red	Red	Red	Red	Green	Red	Red	10
Le, Elizabeth P. V. et al., 2024	Green	Red	Red	Red	Green	Green	Green	Red	Green	Red	Red	Red	Red	Green	Red	Red	9
Li, Zhi-Li et al., 2025	Green	Green	Red	Red	Green	Green	Green	Green	Green	Green	Red	Orange	Red	Green	Red	Red	14
Liu, M et al., 2023	Green	Green	Red	Red	Green	Green	Green	Green	Green	Green	Red	Green	Red	Green	Red	Red	15
Meddings, Zakaria et al., 2024	Green	Red	Red	Red	Green	Green	Green	Red	Green	Red	Red	Red	Red	Green	Red	Red	9
Shan, Dezhi et al., 2023	Green	Green	Red	Red	Green	Green	Green	Red	Green	Red	Red	Orange	Red	Green	Red	Red	12
Shi, Jinglong et al., 2023	Green	Green	Red	Red	Green	Green	Green	Green	Green	Green	Red	Orange	Red	Green	Red	Red	14
Song, Jiajia et al., 2025	Green	Green	Red	Red	Green	Green	Green	Green	Green	Red	Red	Orange	Red	Green	Red	Red	13
Wang, Lingjie et al., 2024	Green	Green	Red	Red	Green	Green	Green	Red	Green	Green	Red	Orange	Red	Green	Red	Red	13
Xia, Hai et al., 2023	Green	Red	Red	Red	Green	Green	Green	Red	Green	Red	Red	Orange	Red	Green	Red	Red	11
Yu, Fan et al. 2025	Green	Green	Red	Red	Green	Green	Green	Green	Green	Green	Red	Orange	Red	Green	Red	Red	14
Zhang, Lianlian et al. 2022	Green	Green	Red	Red	Green	Green	Green	Green	Green	Green	Red	Orange	Red	Green	Red	Red	14
Zhang, Ranying et al., 2021	Green	Green	Red	Red	Green	Green	Green	Green	Green	Red	Red	Orange	Red	Green	Red	Red	13
Zhang, Shuai et al., 2022	Green	Green	Red	Red	Green	Green	Green	Green	Green	Green	Red	Green	Orange	Green	Red	Red	15
Zhang, Xun et al., 2023	Green	Orange	Red	Red	Green	Green	Green	Green	Green	Green	Red	Orange	Red	Green	Red	Red	13

Legend: Green: item completed, orange: item is incomplete, red: item non completed

1. Image protocol quality - well-documented image protocols (for example, contrast, slice thickness, energy, etc.) and/or usage of public image protocols allow reproducibility/replicability.
2. Multiple segmentations - possible actions are: segmentation by different physicians/algorithms/software, perturbing segmentations by (random) noise, segmentation at different breathing cycles. Analyse feature robustness to segmentation variabilities.
3. Phantom study on all scanners - detect inter-scanner differences and vendor-dependent features. Analyse feature robustness to these sources of variability.
4. Imaging at multiple time points - collect images of individuals at additional time points. Analyse feature robustness to temporal variabilities (for example, organ movement, organ expansion/shrinkage).
5. Feature reduction or adjustment for multiple testing - decreases the risk of overfitting. Overfitting is inevitable if the number of features exceeds the number of samples. Consider feature robustness when selecting features.
6. Multivariable analysis with non radiomics features (for example, EGFR mutation) - is expected to provide a more holistic model. Permits correlating/inferencing between radiomics and non radiomics features.
7. Detect and discuss biological correlates - demonstration of phenotypic differences (possibly associated with underlying gene–protein expression patterns) deepens understanding of radiomics and biology.
8. Cut-off analyses - determine risk groups by either the median, a previously published cut-off or report a continuous risk variable. Reduces the risk of reporting overly optimistic results.
9. Discrimination statistics - report discrimination statistics (for example, C-statistic, ROC curve, AUC) and their statistical significance (for example, p-values, confidence intervals). One can also apply resampling method (for example, bootstrapping, cross-validation).
10. Calibration statistics - report calibration statistics (for example, Calibration-in-the-large/slope, calibration plots) and their statistical significance (for example, P-values, confidence intervals). One can also apply resampling method (for example, bootstrapping, cross-validation).
11. Prospective study registered in a trial database - provides the highest level of evidence supporting the clinical validity and usefulness of the radiomics biomarker.

12. Validation - the validation is performed without retraining and without adaptation of the cut-off value, provides crucial information with regard to credible clinical performance.
13. Comparison to 'gold standard' - assess the extent to which the model agrees with/is superior to the current 'gold standard' method (for example, TNM-staging for survival prediction). This comparison shows the added value of radiomics.
14. Potential clinical utility - report on the current and potential application of the model in a clinical setting (for example, decision curve analysis).
15. Cost-effectiveness analysis - report on the cost-effectiveness of the clinical application (for example, QALYs generated).
16. Open science and data - make code and data publicly available. Open science facilitates knowledge transfer and reproducibility of the study.
